# Stimulant medication and suicide mortality in attention-deficit hyperactivity disorder

**DOI:** 10.1192/bjo.2023.643

**Published:** 2024-01-22

**Authors:** Korie M. Rice, Talya Peltzman, Daniel Gottlieb, Brian Shiner, Bradley Vincent Watts

**Affiliations:** Department of Mental Health Services, Veterans Affairs Medical Center, Vermont, USA; Department of Mental Health Services, Veterans Affairs Medical Center, Vermont, USA; Department of Mental Health Services, National Center for Posttraumatic Stress Disorder, Vermont, USA; and Department of Psychiatry, Geisel School of Medicine at Dartmouth, New Hampshire, USA; Department of Mental Health Services, Veterans Affairs Medical Center, Vermont, USA; Department of Psychiatry, Geisel School of Medicine at Dartmouth, New Hampshire, USA; and Office of Rural Health, Veterans Rural Health Resource Center, Vermont, USA

**Keywords:** Attention-deficit hyperactivity disorders, suicide, mortality, epidemiology, statistical methodology

## Abstract

Patients diagnosed with attention-deficit hyperactivity disorder (ADHD) are at an elevated risk for suicide. No prior work has assessed the association between stimulant prescriptions and death by suicide in this population. This retrospective cohort study included Department of Veterans Affairs patients with an active ADHD diagnosis that received stimulant medications between 2016 and 2019. We found that months with active stimulant medication prescription was associated with decreased risk of suicide mortality compared with months without stimulant medication (odds ratio 0.57, 95% CI 0.36–0.88). Our results suggest that prescribing stimulant medications for patients diagnosed with ADHD is associated with decreased risk of suicide mortality.

Suicide is a leading cause of death and major public health concern in the USA.^1^ The aetiology of suicidality remains poorly understood.^2^ However, presence of mental illness remains a frequently cited risk factor for suicide mortality.^2^ Although traditional theories have focused on depression and isolation as primary risk factors for suicide, a growing body of research suggests that disorders characterised by challenges with impulsivity and emotional regulation, such as attention-deficit hyperactivity disorder (ADHD), may also play a role.^2–4^ Of concern, previous research has suggested that patients with ADHD account for approximately 1.5–2 times the rates for suicide attempts and mortality compared with controls.^5^ In the USA, the incidence of ADHD has increased among adults over the past two decades, rising from 9.4 to 13.5 per 10 000 person-years between 2007 and 2016.^6^ Stimulants, the primary medication used to treat ADHD, were prescribed to 6.6% of adults in the USA in 2015 and 2016.^7^

A study by Chang et al found that individuals had lower risk of suicide attempt during months when they were receiving a stimulant medication.^8^ To our knowledge, no study has examined the effects of medication for ADHD on risk of suicide mortality. The objective of our study was to determine if, among individuals diagnosed with ADHD, active stimulant medication treatment is associated with decreased risk of suicide mortality.

## Method

This study was performed in accordance with the Declaration of Helsinki. All procedures involving human participants were approved by the Veteran's Institutional Review Board of Northern New England (approval number 988703). Informed consent was not obtained because this was a retrospective study involving only electronic medical record data.

### Data sources

We collected demographic, diagnostic, pharmacy and healthcare utilisation data from patient electronic health records stored in the Department of Veterans Affairs Corporate Data Warehouse. We identified mortality outcomes with the Department of Veterans Affairs and Department of Defense Mortality Data Repository, which links information on veterans with death certificate data from the Centers for Disease Control and Prevention (CDC) National Death Index.

### Population

We identified Department of Veterans Affairs patients with an ADHD diagnosis between 2016 and 2019, as identified by encounters that included the ICD-10 code F90.^9^ For each patient, we identified the first diagnosis in this period as their index diagnosis. To ensure our population reflected a patient population with active ADHD diagnoses, we censored patients from analysis if they went more than 12 months without a subsequent encounter linked to an ADHD diagnosis.

### Mortality outcomes

Because of the potential problems correctly attributing cause of death, we considered three mortality outcomes: suicide (ICD-10 codes: X60–84, Y87.0, U03), overdose (ICD-10 codes: X40–44, X85, Y10–14) and external-cause mortality (ICD-10 codes: V01–Y36, Y85–87, Y89, U01–03).^10^ The morality outcomes used were not mutually exclusive. The CDC defines external-cause mortality as any accident or injury, either intentional or accidental, that results in death.

### Covariates

We included covariates related to ADHD treatment, patient demographics and healthcare services received. We also included mental health diagnosis burden as defined by patients’ unweighted I-6 index, which is a validated summary measure in which patients receive one point (maximum score of 6) based on the presence of the following DSM categories: substance use disorders, depressive disorder, psychotic disorders, bipolar disorder, trauma disorders and personality disorders.^11^

### ADHD treatment

For each month a patient was characterised as having an active ADHD diagnosis, we assessed whether they had an active stimulant prescription. Stimulant prescriptions in the Department of Veterans Affairs formulary included dextroamphetamine, amphetamine-dextroamphetamine, dexmethylphenidate, methylphenidate and lisdexamfetamine. For a given month, active stimulant prescriptions were identified as any filled prescription whose days’ supply overlapped with the month. Patients had to have at least one active stimulant prescription between 2016 and 2019 to be included in this study.

We characterised the average number of months on versus off stimulants in our study population, as well as demographic characteristics, including gender, race/ethnicity and age as of their index use.

### Analysis

We compared the risk of suicide, overdose and external-cause mortality during months on versus off stimulant prescriptions at a population level. We utilised discreet time-series logistic regression, wherein risk was observed for each month a patient had use, beginning at their index date and continuing until their ADHD diagnosis became inactive, they died or the study period ended. Our analysis compared the risk of mortality during medicated and unmedicated months, adjusting for patient demographic characteristics as well as time-varying covariates, including the number of patient appointments and non-stimulant psychiatric prescriptions during the months of interest. We also included a time-varying covariate summarising patients’ mental health burden in the 12 months before the month of interest. Our models used clustered robust standard errors to account for correlations between months at the patient level.

## Results

The overall cohort included 73 177 Department of Veterans Affairs patients with an active ADHD diagnosis. On average, patients were followed for 29.8 months (s.d. = 14.7) and had an active stimulant prescription fill of 18.5 person-months (s.d. = 14.5) when in the study. The study population represented mostly men and was largely White. The mean age was 39.4 (s.d. = 12.4). Overall, a high percentage of patients had comorbid diagnoses of depression (45.5%) and trauma-related disorders (44.3%) with ADHD (see Supplementary Material available at https://doi.org/10.1192/bjo.2023.643). Patients experienced an increase in out-patient visits for both mental health and non-mental health care during months on stimulant medication. The opposite was true for in-patient stays. Finally, receipt of non-stimulant medication increased across antidepressants, antipsychotics, mood stabilisers, opioids, opioid agonists and sedative anxiolytics during months on stimulant medication (see Supplementary Material).

**Fig. 1:**
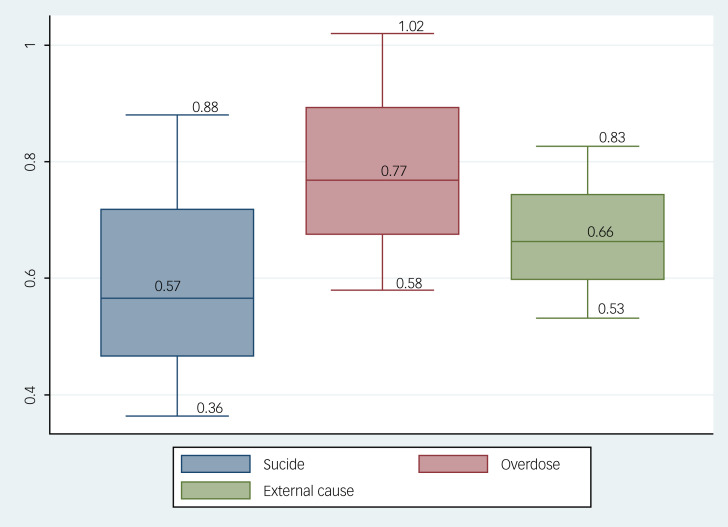
Suicide, overdose and external-cause mortality odds ratios among patients with attention-deficit hyperactivity disorder during months on versus off stimulant medication.

The odds ratio of suicide mortality during months on a stimulant medication was 0.57 (95% CI 0.36–0.88) compared with months off of stimulant medication. Odds ratios for overdoses and external-cause mortality during months on versus off of a stimulant medication was 0.77 (95% CI 0.58–1.02) and 0.66 (95% CI 0.53–0.83), respectively (see Fig. 1).

## Discussion

Among Department of Veterans Affairs patients diagnosed with ADHD, we found that active stimulant medication use was associated with decreased risk of suicide mortality. Our analyses also showed a reduction in risk of overdose and external-cause mortality. This suggests that the findings of reduced suicide mortality are not a result of misclassification of patient deaths.

Building from Chang et al's work, our findings suggest that stimulant medications may reduce the risk of suicide mortality in addition to suicide attempts among patients diagnosed with ADHD. Our study also fits into the broader context of literature showing evidence-based treatment of mental illness may reduce the risk of death by suicide. For example, Watts et al found that evidence-based treatments for opioid use disorder significantly reduce risk of suicide mortality.^12^ Taken together, these findings may suggest that an effective approach to suicide prevention may be providing evidence-based treatment for mental illness rather than specifically targeting suicide alone.

Within our cohort, patients diagnosed with ADHD typically receive more mental healthcare during periods of stimulant treatment. Therefore, it is possible those treatments are either fully or partially responsible for the effect on suicide. It is also unclear if active screening and increased diagnosis of ADHD would yield similar effects. It is also conceivable that the effects of stimulant medication may mediate treatment for depression and substance use disorders among patients with co-occurring ADHD.

In conclusion, our study suggests that treatment with stimulant medications for patients diagnosed with ADHD is associated with a decreased risk of suicide mortality. Replication of our methods are needed among different populations to determine the validity and generalisability of our findings. Future work should seek to better understand ADHD-specific symptoms such as impulsivity, as they relate to suicide risk. For example, replicating this work in a non-veteran population, and an exploratory clinical trial using stimulant medications to treat impulsivity and suicide risk, may be warranted.

## Supporting information

Rice et al. supplementary material 1 Rice et al. supplementary material

Rice et al. supplementary material 2 Rice et al. supplementary material

## Data Availability

Due to data use and privacy constraints, the data used for this work cannot be shared.
