# Supplementary material for: Stimulant medication and suicide mortality in attention-deficit hyperactivity disorder
Source: BJPsych Open. 2024 Jan 22;10(1):e33. doi: 10.1192/bjo.2023.643 (PMC10897683; doi:10.1192/bjo.2023.643)
Supplement: Rice et al. supplementary material 1 — Rice et al. supplementary material [file S2056472423006439sup001.docx]

**Table 1.** Characteristics of study cohort

|  | **Study Cohort**  N=73,177 |
| --- | --- |
|  | mean (SD) |
| Age | 39.37 (12.38) |
|  | %(n) |
| Female | 17.82 (13,043) |
| *Race* |  |
| Asian or Pacific Islander | 2.94 (2,151) |
| American Indian/Alaskan Native | 1.69 (1,239) |
| Black | 8.40 (6,145) |
| White | 86.27 (63,130) |
| Unknown | 0.70 (512) |
| *Ethnicity* |  |
| Hispanic | 8.38 (6,135) |
| Non-Hispanic | 91.39 (66,877) |
| Unknown | 0.23 (165) |
| *Mental Health Diagnoses* | |
|  | mean (SD) |
| Overall* | 1.18 (1.03) |
|  | % (n) |
| Depression | 45.49 (500,681) |
| Bipolar | 8.52 (91,878) |
| Psychosis | 1.56 (15,924) |
| Personality | 3.62 (38,110) |
| Substance | 14.14 (149,542) |
| Trauma | 44.33 (488,075) |

*Summary measure of mental health diagnosis burden in which patients receive one point (maximum score is 6) based on the presence of Diagnostic and Statistical Manual disorder categories.
