# Supplementary material for: Stimulant medication and suicide mortality in attention-deficit hyperactivity disorder
Source: BJPsych Open. 2024 Jan 22;10(1):e33. doi: 10.1192/bjo.2023.643 (PMC10897683; doi:10.1192/bjo.2023.643)
Supplement: Rice et al. supplementary material 2 — Rice et al. supplementary material [file S2056472423006439sup002.docx]

**Table 2.** Characteristics of study cohort during months on and months off prescribed stimulant medication

|  | **Months On Stimulant**  N=1,354,409 | **Months Off Stimulant**  N=823,750 |
| --- | --- | --- |
|  | *Outpatient Visits* | |
|  | mean (SD) | mean (SD) |
| Total | 1.83 (2.49) | 1.49 (3.18) |
| Non-Mental Health | 0.96 (1.55) | 0.82 (1.66) |
| Mental Health | 0.88 (1.55) | 0.67 (2.09) |
|  | *Inpatient Stays per 1000 person-months* | |
|  | mean (SD) | mean (SD) |
| Total | 3.18 (57.8) | 6.88 (86.30) |
| Non-Mental Health | 1.66 (40.72) | 2.72 (52.08) |
| Mental Health | 1.52 (38.91) | 4.16 (64.38) |
|  | *Receipt of Non-Stimulant Medication* | |
|  | % (n) | % (n) |
| Total | 45.01 (609,524) | 30.56 (251,736) |
| Antidepressant | 28.08 (380,229) | 20.51 (168,928) |
| Antipsychotic | 5.58 (75,576) | 4.61 (37,948) |
| Mood Stabilizer | 11.62 (157,427) | 8.68 (71,471) |
| Opioid | 8.18 (110,739) | 4.43 (36,524) |
| Opioid Agonist | 1.72 (23,291) | 1.12 (9,255) |
| Sedative Anxiolytic | 13.86 (187,708) | 6.92 (57,004) |
